# Influence of acoustic cavitation and its combination with H_2_O_2_, acid, and alkali as a pre-treatment technique on lactic acid production from rice as a model food waste^☆^

**DOI:** 10.1016/j.ultsonch.2025.107422

**Published:** 2025-06-07

**Authors:** Zahra Askarniya, Aisha Khan Khanzada, Sławomir Ciesielski, Zongsu Wei, Jacek Mąkinia, Grzegorz Boczkaj

**Affiliations:** a Department of Sanitary Engineering, Faculty of Civil and Environmental Engineering, Gdańsk University of Technology, Poland; b Department of Environmental Biotechnology, University of Warmia and Mazury in Olsztyn, Poland; c Centre for Water Technology, Department of Biological and Chemical Engineering, Aarhus University, Ole Worms Alle 3, 8000 Aarhus C, Denmark; d School of Civil, Environmental, and Architectural Engineering, College of Engineering, Korea University, 145 Anam-ro, Seongbuk-gu, Seoul 02841, Republic of Korea

**Keywords:** Waste management, Circular economy, Sustainability, Ultrasonic cavitation, Bioproducts

## Abstract

Acoustic cavitation was utilized as a pretreatment technique to modify a model food waste (rice) as the substrate of fermentation, enhancing the production of lactic acid. The comparison of a low-frequency (24 kHz) reactor with a high-frequency (120 kHz) reactor demonstrated that the low-frequency reactor remarkably enhanced soluble chemical oxygen demand (sCOD), while the enhancement achieved by the high-frequency one was negligible. Increasing power density from 100 W/L to 400 W/L in the low-frequency reactor showed a continuous rise in sCOD and total lactic acid production. The application of ultrasound at a frequency of 24 kHz and a power density of 400 W/L raised sCOD by 555 %, L-lactic acid by 92 %, D-lactic acid by 43 %, and total volatile fatty acids (VFAs) by 15 %. Acid (HCl) and alkali (NaOH) were combined with ultrasound (24 kHz, 400 W/L) in the pretreatment stage, and they elevated sCOD by 750 % and 625 %, respectively. The pretreatment performed by the combination of ultrasound and acid enhanced L-lactic acid, D-lactic acid, and total VFAs by 71 %, 100 %, and 29 %, respectively. The combination of ultrasound and alkali in the pretreatment stage also increased L-lactic acid and D-lactic acid by 62 % and 104 %, respectively H_2_O_2_ was also combined with ultrasound (24 kHz, 400 W/L) and it raised L-lactic acid and D-lactic acid by 65 % and 109 %, respectively. Microbiological analyses demonstrated that *Limosilactobacillus* sp. formed the highest percentage of species in digestate samples. Economic assessment demonstrated that sole ultrasound (24 kHz and 400 W/L) and its combination with H_2_O_2_ were the most economically viable pretreatment methods, resulting in net savings of €153 and €156 per ton of substrate treatment, respectively.

## Graphical abstract

**Figure:**
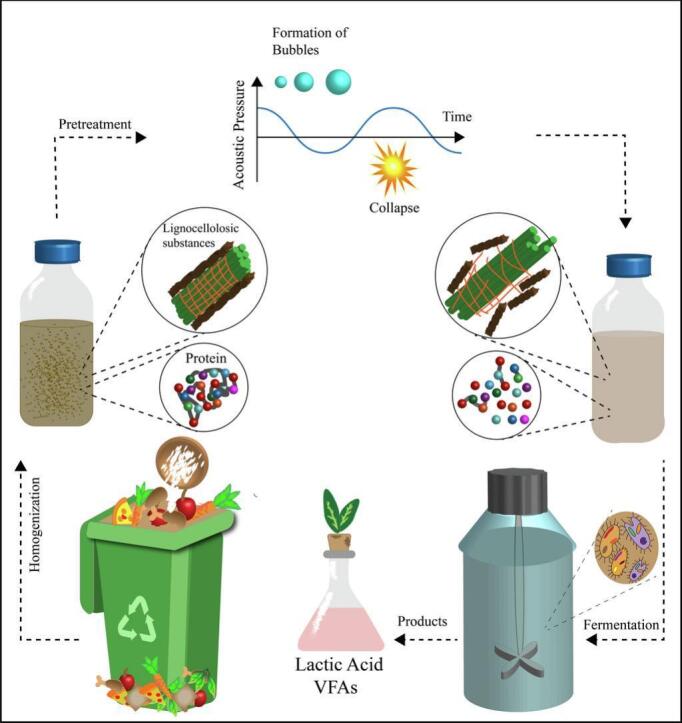

## 1 Introduction

The significant rise in the global population and development of the economy has drastically increased waste production around the world, which has been considered a major global issue in recent years [1]. A global food waste production of 932 million tons per year was estimated by the United Nations [2]. Food waste contains valuable substances, such as proteins, fats, lipids, carbohydrates, and polysaccharides, which can be utilized to produce various products, including biofuels (biomethane, biohydrogen, and biodiesel), volatile fatty acids (VFAs), carboxylic acids, and lactic acid (LA) [3,4]. The production of these valuable compounds through the reuse of waste can cause a reduction in the amount of food waste, supporting circular economy [5].

Anaerobic fermentation is an efficient, cost-effective, and environmentally-friendly way to produce short-chain fatty acids and lactic acid from organic substrates by microorganisms [6,7]. Lactic acid bacteria (LAB) grow well under anaerobic conditions and convert sugars and other fermentable carbohydrates into LA (C_3_H_6_O_3_), a valued substance with various applications like food flavor, food packaging, pharmaceuticals, textile, etc. [[8], [9], [10]]. Typically, the process of biological fermentation of organic material can form a combination of isomers, specifically L-lactic acid (L-LA) and D-lactic acid (D-LA) [11]. In the fermentative approach, the production of LA is performed through the hydrolysis (first stage) and acidogenesis (second stage) stages [12]. Hydrolysis as the first step of fermentation is responsible for increasing the solubility of organic matter consumed by bacteria producing desired products. Therefore, the yield of final products can be unsatisfactory due to a weak hydrolysis stage [4,13,14].

Lignocellulosic food wastes such as rice are resistant to hydrolysis since lignin fraction prevents cellulose molecules from being released [15]. The chemical or physical pretreatment of a substrate can intensify the hydrolysis stage, increasing the solubility of organic matters, and as a result, enhance the availability of nutrients to microorganisms, which can intensify the growth of organisms and the formation of favorable products [5]. For example, cavitation pretreatment techniques are responsible for making a decrease in the size of particles and an increase in surface area, enhancing the hydrolysis stage [[16], [17], [18], [19], [20]]. The generation, growth, and collapse of bubbles in a short duration is known as the cavitation phenomenon [21,22]. The collapse of bubbles can lead to tremendous shear wall stress and high-speed shock waves [23,24]. The force from these shock waves can break nearby solids, such as immersed solids in the liquid, which results in the formation of eddies, enhancing the rate of turbulence contributing to enhanced mass transfer and improved kinetics [25]. Additionally, high local pressure and temperature can appear because of gas and vapor being compressed in the cavities [26]. These extreme temperature and pressure points can lead to the water pyrolysis and the formation of HO^•^, HOO^•^, and H_2_O_2_ [27,28]. All of these effects of cavitation can contribute to the breakage of bonds in heavy molecules and form the lighter ones [[29], [30], [31]]. In acoustic cavitation (AC), the positive and negative pressure responsible for the formation of the cavitation phenomenon is induced through the transmission of ultrasonic waves in the medium [6,28,32].

This study aimed to compare a low-frequency reactor with a high-frequency one as a pretreatment technique to modify the substrate of fermentation. Rice was used as a model food waste since it is consumed as a primary food source for about half of the global population. Approximately 90 % of rice is made of starch [33]. Therefore, it can be useful for the production of LA. The effect of power density on LA and VFAs production was also examined. Additionally, the influence of combining AC with acid, alkali, and H_2_O_2_ as external additives was evaluated in the terms of soluble chemical oxygen demand (sCOD), LA concentration, and VFAs concentration. Microbiology analysis was performed to determine the species community and discuss the roles of each in the production of LA and VFAs. Economic analysis was also performed to determine the economic viability of the pretreatment process. This paper presents a comprehensive study investigating the effect of AC as well as the combination of AC with the additives on the characteristics of substrate affecting the production of lactic acid as a fermentation product, which has been rarely reported in the literature.

## 2 Material and methods

### 2.1 Chemicals, biomass, and inoculum

Hydrogen peroxide (30 %), sodium hydroxide (99 %), and potassium iodide (pure) were purchased from POCH (Poland). 4-chlorobenzoic acid (99 %) was purchased from Thermo Fisher Scientific (USA). Hydrochloric acid (30 %) was bought from Sigma-Aldrich (USA). Rice was purchased from a grocery store in Gdansk, Poland. The rice was boiled and then homogenised to achieve a thick slurry using a Mandine 600 W hand mixer and then, stored at a temperature of 4 °C. For the pretreatment processes, it was mixed with deionized water provided from the Direct-Q® Water Purification System (Merck Millipore, Germany) to provide a substrate loading of 5 % w/v in each experiment. The volatile suspended solid (VSS) of rice before treatment was 16.75 g/L. The inoculum contained a mixture of microbial culture from an anaerobic mesophilic digester, which was taken from a municipal wastewater treatment plant in Gdynia, Poland. Total suspended solid (TSS) and VSS of the inoculum were 30.26 g/L and 23.96 g/L, respectively. To guarantee the efficacy of the inoculum for the fermentation experiments, it was stored at a temperature of 20 °C before utilization.

### 2.2 Pretreatment

**Fig.1:**
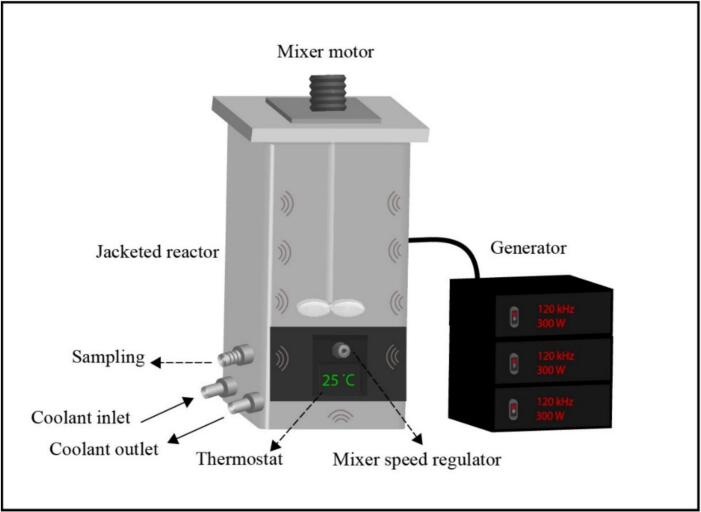
The scheme of the high-frequency AC reactor working at a frequency of 120 kHz.

Two kinds of AC reactors were utilized for the pretreatment of rice before the fermentation. One experiment was done in a self-designed AC reactor which worked at a high frequency of 120 kHz. The scheme of this reactor is observed in Fig. 1.

The shape of the reactor is rectangular with dimensions of 280 mm height and 130 mm width. The reactor is equipped with a mixer and set of transducers generating the ultrasounds. The transducers were installed in the walls of this reactor. A volume of 3 dm^3^ and power of 900 W (3 generators, each providing a power of 300 W) providing a power density of 300 W/L were employed for this experiment.

**Fig.2:**
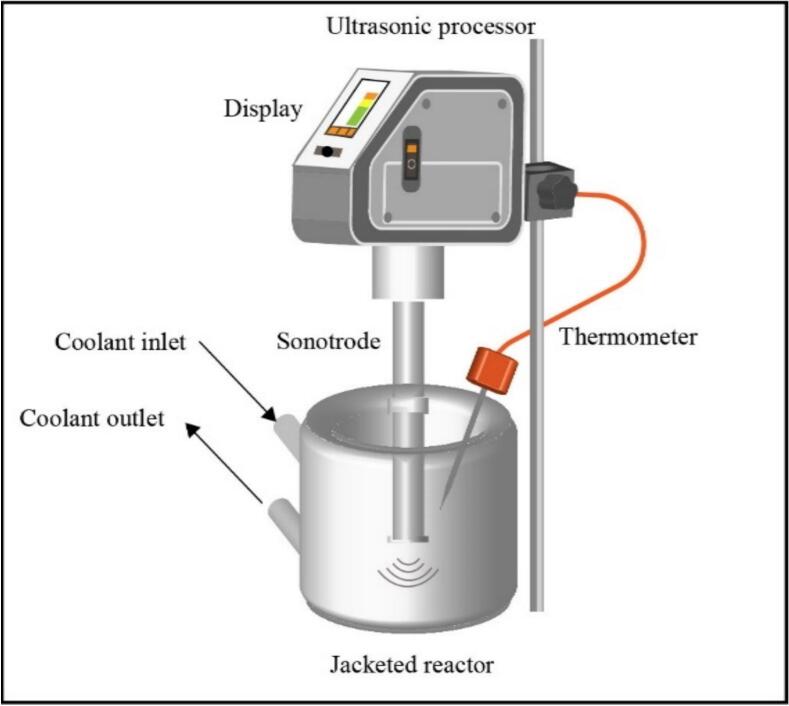
The scheme of the low-frequency AC reactor working at a frequency of 24 kHz.

Other pretreatment experiments were accomplished in a low-frequency reactor (Hielscher 400th) equipped with a sonotrode with an immersion depth of 45 mm, a diameter of 14 mm, and a radiating surface of 2.2 cm^2^ (s24d14D). The scheme of the low-frequency reactor is demonstrated in Fig. 2.

This reactor worked at a fixed frequency of 24 kHz. The volume of this rector was 500 ml and power densities of 100 W/L, 300 W/L, and 400 W/L were investigated in this reactor. This range of power density was selected based on the suitable range reported in the earlier review paper [27]. In the both reactors, cooling water passing through a cooling coil was responsible for keeping the temperature constant at 25 ± 3 °C during the pretreatment.

After the selection of the ideal reactor and power density, AC was combined with HCl (pH 3), NaOH (pH 11), and H_2_O_2_ as external additives to investigate the effect of these combinations on the final results.

### 2.3 Batch fermentation

Batch fermentation was performed to produce LA and VFAs from rice as the substrate which was pretreated by AC under different conditions. The fermentation reactors were 500 ml flasks, agitated at 100 rpm with an inoculum substrate ratio (ISR) of 1:4 while maintaining a constant temperature of 35 °C throughout the fermentation duration. The initial pH of all samples was 7 at the start of fermentation but it was uncontrolled during the process. Samples were collected daily for 5 days to monitor the fermentation dynamics over time, followed by centrifugation for 30 min at 10000 rpm. Subsequently, the liquid portion was divided from the solid component. For additional analysis, the filtration of separated supernatant was performed by the application of membrane filters with a 0.45 µm pore size. This analysis involved measuring the levels of sCOD, L-LA, D-LA, and VFAs (acetic acid, propionic acid, butyric acid, and valeric acid).

### 2.4 Analytical methods

A high-performance liquid chromatography (HPLC) with UV detection (monitored wavelength 254 nm) using a Hitachi LaChrom Elite system was applied for the quantification of LA. This HPLC system had an Astec (Sigma Aldrich) CLC-D column (150 mm × 4.6 mm, 5 µm). The mobile phase (flowrate: 1 mL/min) contained a 5 mM CuSO_4_ solution in deionized water. Samples (10 µL injection volume) were injected by autosampler.

VFAs concentration was analysed using a gas chromatography (GC; Shimadzu Nexis GC-2030 instrument) with a flame ionization detector (FID), which was equipped with an Agilent J&W DB-FFAP column (30 m, 0.25 mm ID, 0.25 µm film thickness). Samples were injected by means of autosampler (1 µL injection volume). The temperatures of the injector and detector were 250 °C. Temperature program: 50 °C (hold 5 min.) – ramp 5 °C/min. to 120 (hold 5 min.) – ramp 25 °C/min to 200 °C (hold 20 min.).

UHPLC Nexera XS system (Shimadzu, Japan) with a photo diode array detector (SPD-M40) was employed for the detection of *para*-chlorobenzoic acid (p-CBA), which was used as a probe compound for hydroxyl radicals, monitored at 239 nm. This HPLC was equipped with a chromatographic column (model: Zorbax C-18, 150 mm × 4.6 mm, 3.5 µm, Agilent, USA), and the mobile phase contained 10 mM phosphoric acid and methanol with a ratio of 45:55 (v/v). The flow rate was set at 1.0 mL/min, and the oven temperature maintained at 30 °C. Samples were injected (10 µL injection volume) by an autosampler (SIL-40C XS). A calibration curve was generated using a standard solution of p-CBA dissolved in deionized water.

A UV-1900i spectrophotometer (Shimadzu, Japan) was used to detect I_3_^−^ at a wavelength of 352 nm, as a demonstration of oxidation of potassium iodide by hydroxyl radicals generated in the high-frequency and low-frequency reactors.

TSS and VSS levels were determined following standard procedures suggested by the American Public Health Association [34]. pH test strips (Merck, Germany) were used for pH measurement. sCOD was measured by the utilization of Hach-Lange testing vials LCK 214 (0–1000 mg O_2_) and LCK 914 (5–60 g/L O_2_). After the addition of the samples to the vials, they were digested using a high speed digestion system (HACH HT200S). Once cooled, sCOD was assessed with the Hach-Lange DR3900 spectrophotometer.

### 2.5 Microbiological analyses

The composition of microorganisms was analysed in the sample of activated sludge (inoculum) and four digestate samples which were different in external additives. The immediate freezing (20° C) of biomass was performed after sampling. FastDNA Spin kit for soil (MP Biomedicals, USA) was utilized for DNA extraction. Thawing of samples happened at room temperature. Phosphate buffer was used for the re-suspension of 200 mg of semi-dry biomass. The maximum speed in a Uniequip device (Uniequip, Planegg, Germany) was applied for bead beating in 2 min [35]. Agarose gel electrophoresis was employed for the evaluation of the quality of the extracted DNA. Quant-iT BR DNA Assay (Thermo Fisher Scientific, USA) was used to fluorometrically measure the DNA concentration.

The 16S Barcoding Kit (SQK-16S114.24) (Oxford Nanopore Technologies) was utilized for the preparation of 16S-meta library via MinION (Oxford Nanopore Technologies). LongAmp 2X Master Mix (New England Biolab, Herts, UK)  was added to the DNA, and then, it was barcoded with 16S primers provided by a kit according to the manufacturer’s guideline. The amplification was performed by Eppendorf Mastercycler (Eppendorf, Hamburg, Germany) according to the following PCR conditions: 1 cycle of initial denaturation at 95 °C in 1  min, 30 cycles of denaturation at 95 °C in 20 sec, annealing at 55 °C in 30 sec, and extension at 65 °C in 2  min, followed by 1 cycle of final extension at 65 °C in 5  min. R10.4.1 (FLO-MIN114) was the flow cell applied for the analysis. The run was live-base called employing Guppy v3.2.10 which is included in the MinKNOW v5.8.7 (v23.11.5) software (Oxford Nanopore Technologies). The EPI2ME platform (Oxford Nanopore Technologies; https://epi2me.nanoporetech.com) supplies *16S rRNA* workflow, which was applied for demultiplexing and bacterial taxonomy assignment to the taxonomic genus level, according to the National Centre for Biotechnology Information (NCBI) *16S rRNA* bacterial database [36]. The sequencing reads were filtered to a minimum Q-score of 9, followed by classification to bacterial phylum and genus level.

## 3 Results and Discussion

Parameters including frequency and power density can influence the performance of AC as a pretreatment method to modify the substrate leading to an enhancement in the production of LA and VFAs through fermentation. The effect of these parameters was discussed in the following paragraphs.

### 3.1 High-frequency and low-frequency reactors

Cavitation can be used as a method for the pretreatment of the substrate of fermentation due to its mechanical and chemical effects [37]. These effects can depolymerize complex heavy molecules like starch in rice and form simpler ones like glucose. To modify the solubility of the substrate demonstrated by an increase in sCOD, two different reactors working at a high frequency of 120 kHz and low frequency of 24 kHz were employed at a constant power density of 300 W/L. Since energy input primarily affects the cost of treatment, the goal was to determine which reactor is more suitable when operating at the same cost.

Hydroxyl radicals generated from the pyrolysis of water molecules through hot spots (Eq. (1) are considered the chemical effect of this phenomenon [38,39].

(1) H_2_O +AC → ^•^OH + ^•^H

This reactive radical might be able to break the side chains of large molecules such as lignin, resulting in the depolymerization and subsequent production of simpler ones [40]. Furthermore, the hydroxylation of the molecules has the potential to increase the hydrophilicity of those molecules and as a result, can increase their solubility [41]. Therefore, it might increase the availability of organic matter for the consumption by microorganisms.

**Fig. 3:**
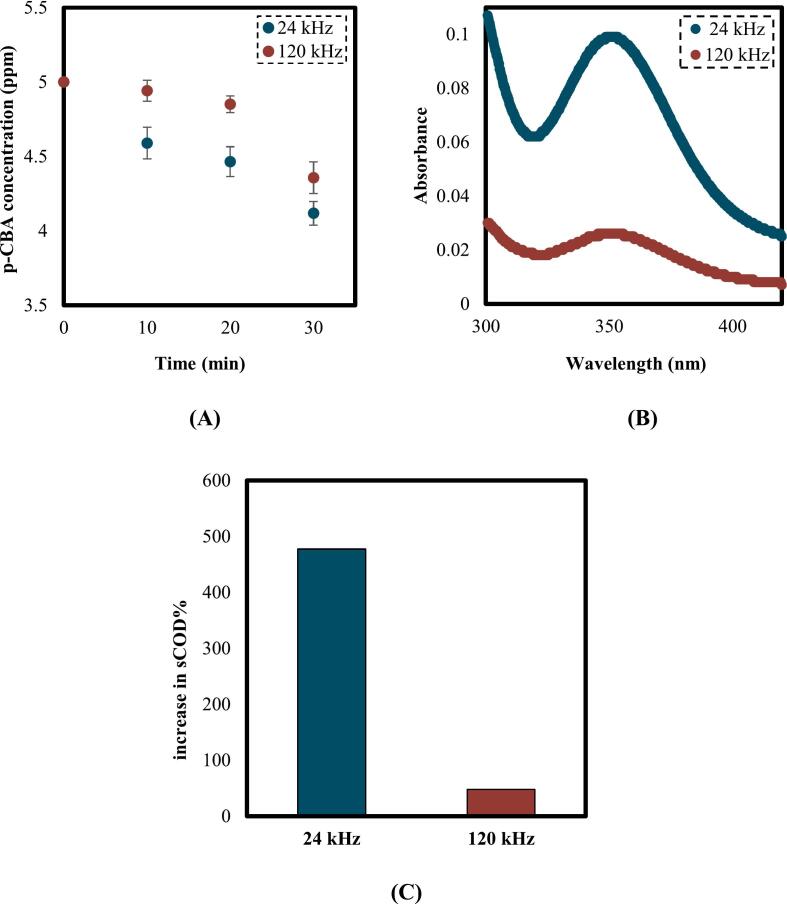
A) Degradation of p-CBA in the high-frequency (120 kHz) and low-frequency (24 kHz) reactors. B) Spectrophotometer results for the absorbance of I_3_¯. C) Increase in sCOD made in the high-frequency and low-frequency reactors (power density: 300 W/L, substrate loading: 5 % w/v, AC duration: 30 min).

To compare the generation of hydroxyl radicals in these two reactors, *para*-chlorobenzoic acid (p-CBA) was utilized since this compound is known as a probe compound for hydroxyl radicals screening as it reacts with this radical at a high second-order rate constant of 5 × 10^9^ M^−1^ s^−1^ [42,43]. Hence, a decrease in the concentration of this compound during cavitation processes can be a demonstration of the generation of hydroxyl radicals. A concentration of 5 ppm of p-CBA was performed in the high-frequency and low-frequency reactors at a power density of 300 W/L in a process time of 30 min, and the results are shown in Fig. 3 **A**.

As it is observed, the concentration of p-CBA decreased from 5 ppm to 4.12 ppm in the low-frequency reactor and 4.36 ppm in the high-frequency reactor. Additionally, to confirm the formation of hydroxyl radicals, a 5 mM potassium iodide (KI) solution was also employed. In the presence of hydroxyl radicals and iodide ions (I^−^), the following reactions are likely to occur [44].

(2) ^•^OH + I^−^ → I + HO^−^

(3) I + I^−^ → I_2_^−^

(4) 2I_2_^−^ → I_2_ + 2I^−^

(5) I_2_ + I^−^ → I_3_^−^

The oxidation of iodide ions by hydroxyl radicals and the subsequent generation of I_3_^−^ were confirmed by the color change to light yellow at the end of the experiment, analyzed by the spectrophotometer. The result of UV absorbance of I_3_^−^ at 350 nm is observed in Fig. 3 **B**. According to the results, the low-frequency reactor generated higher amount of hydroxyl radicals.

The enhancement in sCOD achieved by these two reactors is demonstrated in Fig. 3 **C**. As observed, the low frequency reactor made a remarkable enhancement in the sCOD (477 %), while the effect of high-frequency reactor was negligible (48 %).

In addition to the different amount of generated hydroxyl radicals, another reason for this large difference in sCOD enhancements can be the different magnitude of mechanical effects in these two reactors as reported in the literature [45,46].

Low-frequency provides a longer time for the growth of bubbles leading to bubbles with larger sizes. The implosion of these large bubbles generates a high level of energy resulting in the harsher mechanical effects of cavitation. The extreme collapse of these large bubbles creates strong shock waves [47]. This collapse also produces high shear forces, with pressures around 3.5 kPa, transferring energy quickly and intensely to the surrounding area [48]. These harsh mechanical conditions can break chemical bonds in organic materials, causing their direct disintegration [29]. Therefore, these mechanical effects might have the ability for the breakage of complex carbohydrates such as starch and the formation of simple ones with higher solubility [27]. The increase in sCOD in low-frequency reactor can be related to the potential of low-frequency cavitation to depolymerize cellulose leading to a decrease in particle size and a rise in solubility, which as a result, can intensify the interaction between nutrients and microorganisms [16]. Therefore, the low-frequency reactor was selected as the pretreatment stage for the rest of the experiments.

### 3.2 Evaluation of ultrasound power density

Power density can have an extreme effect on the performance of cavitation as a pretreatment method for fermentation processes. Three different power densities of 100 W/L, 300 W/L, and 400 W/L were investigated to determine the effect of this factor on sCOD, and the subsequent production of LA and VFAs. As observed in Table 1, an increase in the power density has resulted in a continuous increase in sCOD.

**Table 1:** Effect of power density on sCOD at a frequency of 24 kHz, substrate loading of 5 % w/v, and AC duration of 30 min.

**Pretreatment Process**	***Power density (W/L)***	**VSS (g/L)**	**Increase in sCOD%**
AC	***400***	14.99 ± 0.12	555 ± 17
AC	***300***	15.01 ± 0.04	477 ± 24
AC	***100***	15.43 ± 0.17	82 ± 5

Hydrolysis can severely restrict the biochemical formation of valuable products from lignocellulosic food wastes [13]. Cavitation as a pretreatment technique has the potential to mitigate this restriction. Lower power densities might be insufficient for the breakage of chemical bonds of starch in rice to result in a remarkable increase in its solubility. Increasing the power density till an optimum value can intensify the activity of cavitation [49,50]. It is worth mentioning that too high power density can cause cavity clouds, leading to a drastic decrease in cavitation activity, hence, it is better to find an optimum value of power density [51]. In the current research, it was found that along with increasing power, the effectiveness of the process also increased in the case of sCOD enhancement. Thus, further optimization should include additional criteria, mainly the economic one.

**Fig. 4:**
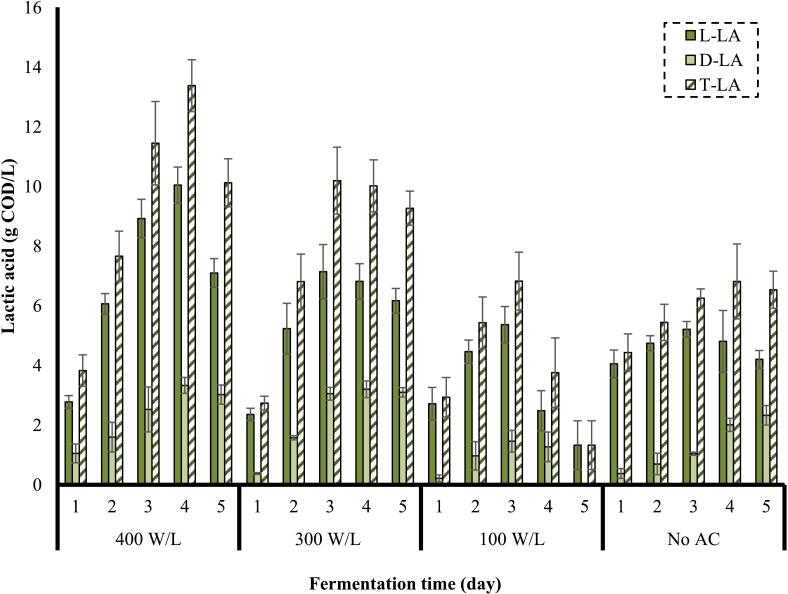
Effect of power density on the concentration of LA (frequency: 24 kHz, substrate loading: 5 % w/v, AC duration: 30 min).

In Fig. 4, the effect of different power densities on the concentration of LA is observed.

As observed in Fig. 4, L-LA was produced more than D-LA during the fermentation primarily due to the metabolic pathways of LAB. The preference for L-LA production might be influenced by the fermentation environment, which generally supports the growth of homofermentative bacteria that predominantly produce the L-isomer [52]. Moreover, these bacteria have evolved to favor L-LA because it is less toxic to cells compared to D-LA, thereby offering a survival advantage in natural and industrial fermentation processes [53].

Under the frequency of 24 kHz, substrate loading of 5 % w/v, and AC duration of 30 min, an increase in the power density in the pretreatment stage caused a continuous enhancement in the concentration of L-LA through fermentation. The intense collapse of cavitation bubbles generated at high power densities facilitates the disintegration of biomass, thereby enhancing microbial activity by providing more available organic matters for their consumption and the overall fermentation process [5,54]. A maximum concentration of L-LA (10.0 g COD/L) was obtained from the substrate pretreated at a power density of 400 W/L observed on the 4^th^ day of fermentation. L-LA concentration achieved from the substrates pretreaed at power densities of 300 W/L and 100 W/L peaked at 7.1 g COD/L and 5.3 g COD/L, respectively, which both were observed on the 3^rd^ day of fermentation. The maximum concentration of D-LA obtained from the substrate pretreated by AC at a power density of 300 W/L (3.7 g COD/ L) was a little higher than the maximum concentration achieved from the substrate pretreated at a power density of 400 W/L (3.3 g COD/L), which both happened on the 4^th^ day of fermentation. The highest concentration of D-LA produced from the substrate pretreated at a power density of 100 W/L was 1.5 g COD/L. The LA concentrations achieved by the control process (fermentation without pretreatment, No AC) also peaked at 5.2 g COD/L (L-LA) and 2.3 g COD/L (D-LA), respectively. A total LA (T-LA), demonstrated in Fig. 4**,** highlights a similar trend as L-LA due to the dominance of L-LA over D-LA.

Acetic acid, propanoic acid, and butyric acid are in the category of VFAs which can be formed through anaerobic fermentation by acetogenic bacteria based on the following reactions.

(6) C_6_H_12_O_2_ + H_2_O_2_ → 4H_2_ + 2CH_3_COOH + CO_2_

(7) C_6_H_12_O_2_ + 2 H_2_ → 4H_2_ + 2CH_3_CH_2_COOH + CO_2_

(8) C_6_H_12_O_2_ + H_2_O_2_ → 2H_2_ + 2CH_3_CH_2_CH_2_COOH + CO_2_

**Fig. 5:**
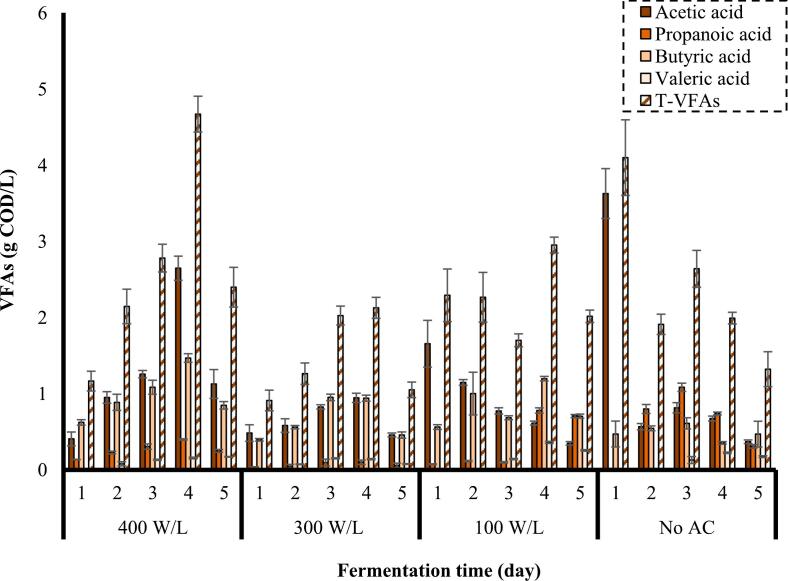
Effect of power density on the concentration of VFAs (frequency: 24 kHz, substrate loading: 5 % w/v, AC duration: 30 min).

VFAs are valuable products utilized as raw substances for the generation of biodegradable thermoplastics as well as for the intensification of biosynthesis value of polyhydroxyalkanoates [55,56]. The effect of different power densities on the generation of VFAs was investigated and the results are demonstrated in Fig. 5**.**

As shown in Fig. 5, the maximum concentration of VFAs (4.7 g COD/L) was obtained from the substrate pretreated by AC at a power density of 400 W/L observed on the 4^th^ day of fermentation. Total VFAs (T-VFAs) of 4.1 g COD/L was also produced by the control process (No AC) on 1^st^ day. The concentration of T-VFAs generated from the substrates pretreated by AC at power densities of 300 W/L and 100 W/L peaked at 2.1 g COD/L (4^th^ day) and 2.9 g COD/L (4^th^ day), respectively. It is observed that no valeric acid was produced on the 1^st^ day of fermentation in all of the studied conditions. Acetic acid and butyric acid were dominant products of VFAs produced from substrate pretreated by AC. However, in the case of the control process and the substrate pretreated at a power density of 100 W/L, the concentration of propanoic acid was also considerable. It is worth mentioning that the maximum concentrations of acetic acid (3.6 g COD/L) and propanoic acid (1.1 g COD/L) were obtained by the control process on 1^st^ and 3rd day of fermentation, respectively. It was hypothesized that the content of substrate after modification by AC might have favored LAB dominance, which could hinder microorganisms responsible for VFAs production due to the unfair competition for sugars, resulting in an enhancement in LA concentration but a reduction in VFAs production compared to the control process. However, in the case of the substrate pretreated at the highest power density of 400 W/L provided higher sCOD, the competition between organisms might have been easier because of the excess amount of soluble organic substances. Therefore, the production of both LA and VFAs increased compared to the control process.

A comparative analysis between the outcomes of the current study and some data reported in the literature is demonstrated in Table 2. All of the mentioned studies have utilized direct-contact ultrasonic probes as an ultrasound generator. The highest increase in total LA concentration reported in the literature was 62 % while the maximum increase in total LA for this study was 95 %. Similarly, the highest increase in the sCOD of food waste-based substrates reported in the literature was drastically lower than the sCOD increase obtained in this study, which can be related to various parameters including the content of food waste, geometry, efficiency of AC reactor, etc.

**Table 2:** Comparison made in the effect of acoustic cavitation on the production of LA and VFAs through the fermentation of food-based substrate between this study and literature.

**Substrate type**	**Frequency (kHz)**	**Power** **density (W/L)**	**AC time (min)**	**SCOD increase**	**Increase in LA or VFAs**	**Ref.**
***Rice***	***24***	***400***	***30***	***555 %***	***95 % (LA)*** ***15 % (VFAs)***	***This study***
Food waste	28	3200	30	−	62 % (LA)	[57]
Stillage after bioethanol production on wasted bread	20	−	10	−	15 % (LA)	[8]
Food waste	20	147	24	9 %	7 % (VFAs)	[58]
Food waste	−	837	30	22.1 %	70 % (VFAs)	[59]
Mixture of rice, cabbage, pork, tufo	20	480	15	209 %	58 % (VFAs)	[60]
Mixture of bread, tea wastes, potato peels, rice, banana peels	20	232	30	159 %	No increase (VFAs)	[15]
Food waste	20	147	24	9 %	36 % (VFAs)	[61]

### 3.3 Enhancing pretreatment performance by external additives

After the experiments accomplished for the selection of the ideal reactor and the suitable power density, more experiments were performed as an attempt to enhance the formation of desired products. This attempt was based on the combination of AC and external additives: 1) acid (HCl) or alkali (NaOH) for the adjustment of pH, 2) hydrogen peroxide (H_2_O_2_) as an oxidant. The addition of acid and alkali to the pretreatment stage was demonstrated through a change in pH (HCl: pH 3, NaOH: pH 11, No additive: pH 7). It should be mentioned that the pH was different just in the pretreatment stage done by AC and later, it was adjusted to 7 before the start of fermentation. Fermentation was performed at an initial pH of 7 in all experiments.

Microbiological tests were performed to analyze the community of species in the samples pretreated by the combination of AC and external additives to determine the role of this community in the formation of desired products.

#### 3.3.1 Changes in the microbial community

Oxford Nanopore Technologies based on the entire *16S rRNA* gene was employed for the analysis of the bacterial community in the inoculum and fermented samples. A total of 555,850 filtered reads were obtained (minimum: 55298; maximum: 197701). The analysis showed different bacterial community structures in the analyzed samples. The community of microorganisms observed in the inoculum was remarkably more diverse than the ones seen in the digestate samples. The inoculum contained the highest effective number of species (99.4). However, in the case of digestate samples, the effective number of species was in the range from 7.9 (pH 7) to 10.2 (AC + H_2_O_2_). The Shannon diversity index was also maximum in the inoculum (4.6). In the digestate samples, the Shannon index values were 2.1 (pH 7 and pH 3), 2.2 (pH 11), and 2.3 (AC + H_2_O_2_).

**Fig. 6:**
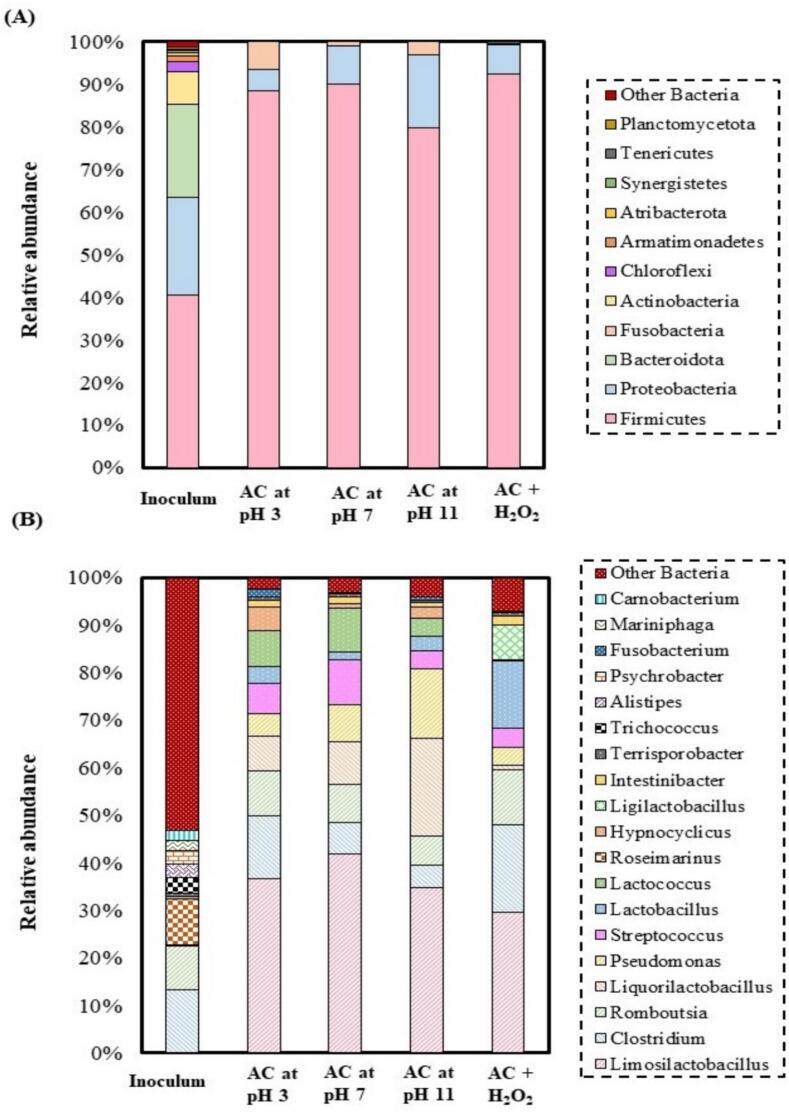
Average relative abundance (%) of the sequences identified in the digestate samples pretreated by AC. Other Bacteria category includes unidentified taxon and taxon with relative abundance lower than 0.5 % (A: phylum level, B: genus level).

As observed in Fig. 6 **A**, at the phylum level, the main components of the inoculum sample were *Firmicutes* (40.43 %), *Proteobacteria* (23.06 %), *Bacteroidota* (21.88 %) and *Actinobacteria* (7.43 %). The proportion of other phyla was less than 5 %. *Firmicutes* showed the highest abundance in the digestate samples. Almost the same numerous members of *Firmicutes* were found in the digestate samples. However, the percentage of this phyla found in the sample pretreated by AC at pH 11 was the lowest. The numerous members of the phyla were 92.53 % in the sample pretreated by AC + H_2_O_2_, 90 % in the sample pretreated by AC at pH 7, 88.49 % in the sample pretreated by AC at pH 3, and 79.85 % in the sample pretreated by AC at pH 11. Bacteria from the *Proteobacteria* phylum were less numerous. Their frequency increased from 5.07 % in the sample pretreated by AC at pH 3 to 16.96 % in the sample pretreated by AC at pH 11. In the sample pretreated by AC + H_2_O_2_, 6.79 % *Proteobacteria* were detected. The members of *Fusobacteria* were 1.05 % and 6.37 % in the samples pretreated by AC at pH 3 and pH 11, respectively, and it was negligible in the samples pretreated by AC at pH 7 and AC + H_2_O_2_.

As displayed in Fig. 6 **B**, at the genus level, the microbial community structures in the digestate samples are almost similar, especially in the samples pretreated by AC at pH 3, pH 7, and pH 11.

In the inoculum, more than 53 % of all genera were rare or unidentified. Numerous genera showed an abundance of more than 2 %. Among them, *Clostridium* sp*.* (13.18 %), *Romboutsia* sp. (9.35 %), *Trichococcus* sp*.* (3.22 %), A*listipes* sp*.* (2.87 %), *Psychrobacter* sp. (2.75 %), *Mariniphaga* sp. (2.07 %) and *Carnobacterium* sp. (5.0 %) were the most abundant in this sample. In the digestate samples, the structure was similar with regard to the dominant bacteria. Bacteria of the genus *Limosilactobacillus* sp*.* (mean value 35.71 ± 5.10 %) were most frequently represented. In addition, the following genera were represented in large numbers: *Clostridium* sp. (mean 10.73 ± 6.34 %), *Romboutsia* sp. (mean 8.82 ± 2.30 %), *Liquorilactobacillus* sp. (mean 9.41 ± 8.23 %), *Pseudomonas* sp. (mean 7.66 ± 4.94 %), *Streptococcus* sp. (mean 5.97 ± 2.66 %), *Lactobacillus* sp. (mean 5.59 ± 5.87 %) and *Lactococcus* sp. (mean 5.21 ± 4.09 %). The mean abundance of the other genera was below 0.5 %. The most abundant LAB − *Limosilactobacillus* sp. − is known as a heterofermentative LA bacterium and has shown a high ability to degrade aldehyde compounds. The highest percent of *Limosilactobacillus* sp. was found in the sample pretreated with AC at pH 7 (no additive), and the lowest was found in the sample pretreated by AC + H_2_O_2_. The high abundance of this bacterium in all treatment variants suggests that it must play an important role in biomass conversion. The abundance of some bacteria (e.g. *Liquorilactobacillus* sp., *Pseudomonas* sp.) increased in the digestate samples pretreated at higher pH in the pretreatment stage, while some others decreased (e.g. *Clostridium* sp., *Romboutsia* sp.). The total abundance of LAB was very similar in all digestate samples, ranging from 67.81 % in the sample pretreated by AC at pH 11 to 71.44 % in the sample pretreated by AC + H_2_O_2_.

The abundance of *Lactobacillus* sp. and *Ligilactobacillus* sp. was significantly higher in the sample pretreated by AC + H_2_O_2_. Another LAB with high abundance is *Liquorilactobacillus* sp. which belonged to the large genus *Lactobacillus* sp. before reclassification. Interestingly, its abundance increased in the digestate samples pretreated at higher pH in the pretreatment stage, while it was significantly lower in the sample pretreated by AC + H_2_O_2_. It seems that the presence of H_2_O_2_ led to a displacement of *Liquorilactobacillus* sp. By the other LAB – *Lactobacillus* sp. and *Ligilactobacillus* sp.

Another bacterium that increased in the presence of H_2_O_2_ is *Clostridium* sp., which has the potential to produce LA [62]. This bacterium produces short-chain fatty acids and many metabolic end substances through the consumption of carbohydrate and fermentation of amino acid, and its presence is desirable for LA production [63].

#### 3.3.2 Evaluation of the combination of AC with acid and alkali in the pretreatment stage

Diluted acid pretreatment often requires high temperatures (above 100 °C) and extended reaction times, which can cause the formation of inhibitory byproducts. Similarly, alkaline pretreatment generally demands high temperatures (100–180 °C). The combination of acid or alkaline pretreatments with AC can enable the process to occur under milder conditions, reducing the need for extreme temperatures and minimizing byproduct formation [[64], [65], [66]]. The combination of AC and HCl or NaOH can increase the solubility of organic matter and afterwards, contribute to an enhancement in the formation of suitable products. The influence of the combination of these materials with AC on sCOD was investigated and the results are observed in Table 3.

**Table 3:** Effect of combination of AC with acid and alkali in the pretreatment stage on sCOD at a frequency of 24 kHz, power density of 400 W/L, substrate loading of 5 % w/v, and AC duration of 30 min.

**Pretreatment process**	***Pretreatment pH***	**VSS (g/L)**	**Increase in sCOD%**
AC + HCl	***3***	14.74 ± 0.15	750 ± 37
AC	***7***	14.99 ± 0.12	555 ± 17
AC + NaOH	***11***	14.86 ± 0.04	625 ± 23

AC combined with both acid and alkali has increased sCOD, compared to AC in the neutral condition. In the case of the acidic condition, the reason behind the increase in sCOD is the effectiveness of acid for the breakage of glycoside bonds in starch molecules and therefore, the generation of simpler molecules with higher solubility [67]. Alkaline pH might be capable of weakening the structure of starch, increasing its vulnerability to cell disruption performed by AC [45,68]. The alkali-induced ionization of hydroxyl groups can enhance the mobility of starch, resulting in easier leaching [33]. Furthermore, the application of AC in combination with alkaline or acidic pretreatment can enhance the removal of hemicellulose and lignin by generating microturbulence and shear forces. Hence, it can facilitate more effective diffusion and interaction between the chemical reagents and the lignocellulosic matrix, and in overall increase the mass transfer and kinetics. Since hemicellulose and lignin form a protective network around cellulose, their removal increases cellulose accessibility to enzymatic attack and subsequently improves hydrolysis efficiency [64,66]. Therefore, it can be noted that both acid and alkaline in combination with AC could intensify the disintegration of starch in the pretreatment stage and lead to a higher solubility demonstrated through higher sCOD.

**Fig. 7:**
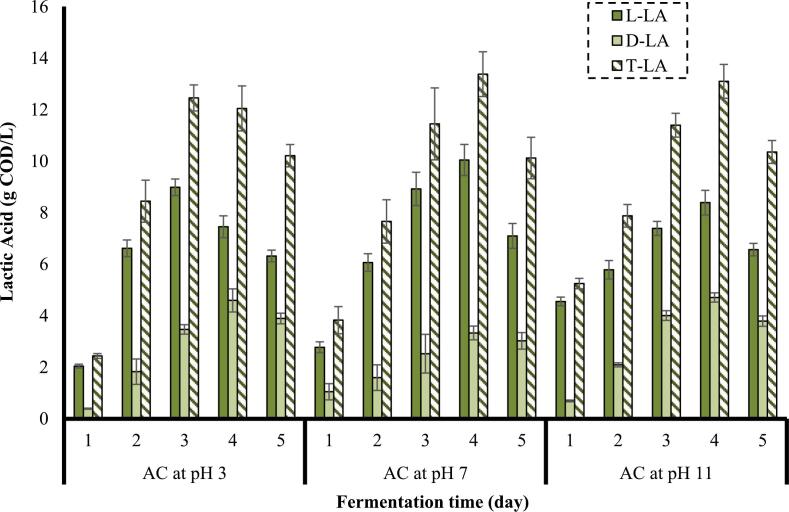
Effect of the combination of AC with acid and alkali in the pretreatment stage on the concentration of LA (frequency: 24 kHz, power density: 400 W/L, substrate loading: 5 % w/v, AC duration: 30 min).

As shown in Fig. 7, pretreatment performed by AC at a pH of 7 (no additives) later resulted in the maximum concentration of L-LA (10.0 g COD/L) among the investigated pH in the pretreatment stage. The concentrations of L-LA achieved by the substrates pretreated at pH 3 and pH 11 peaked at 8.9 g COD/L and 8.4 g COD/L, respectively. The soluble substances formed through the combination of AC with the acid and alkali might have led to a displacement of some portion of *Limosilactobacillus* sp. with other bacteria. The highest percent of L-LA achieved by the substrate pretreated by AC at pH 7 (no additive) might be due to the highest percent of *Limosilactobacillus* sp. as an adaptable LAB [69]. Furthermore, *Streptococcus* sp. and *Lactococcus* sp.*,* which just produce L-LA but not D-LA, had the highest percentage in this substrate [70]. It can be another reason for the highest amount of L-LA produced in this substrate.

The substrate pretreated by AC combined with the acid and alkali in the pretreatment stage resulted in the maximum concentration of the D-LA (4.6 g COD/L and 4.7 g COD/L, respectively), observed again on the 4^th^ day of fermentation. The amount of D-LA achieved by a pH of 7 peaked at 3.3 g COD/L. The higher value of D-LA can be attributed to the higher percentage of *Lactobacillus* sp. in the substrate pretreated by AC at pH 3 and pH 11, since *Lactobacillus* sp. is capable of producing D-LA, while some species such as *Lactococcus* sp. produce just L-LA [70]. According to the results illustrated in Fig. 7, there is a negligible difference between the maximum T-LA obtained by substrate pretreated by AC at pH 7 and pH 11, and the maximum T-LA achieved by substrate pretreated by AC at pH 3 is slightly lower than the others.

It should be taken into account that there might have been a maximum capacity of nutrients which could be consumed by microorganisms in order to process in a specific time, and the provision of more nutrients had no effect and did not increase the productivity [71]. Additionally, there is a maximum concentration of products which can be produced by microorganisms, and above that value, the inhibitory effect of products is possible as the products concentration higher than a specific value can be toxic for microorganisms [72]. Hence, higher sCOD values provided by AC under acidic and alkaline conditions caused no increase in the yield of T-LA.

**Fig. 8:**
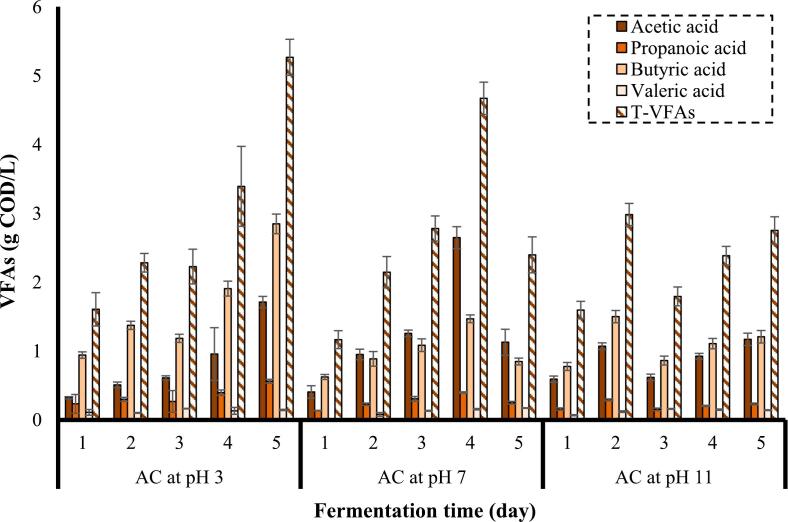
Effect of combination of AC with acid and alkali in the pretreatment stage on the concentration of VFAs (frequency: 24 kHz, power density: 400 W/L, substrate loading: 5 % w/v, AC duration: 30 min).

The amount of VFAs produced by the substrates pretreated using AC combined with acid and alkali on the production of VFAs is presented in Fig. 8.

The concentration of VFAs produced from the pretreated substrate by AC under acidic, neutral, and alkaline conditions shows the peak of T-VFAs at 5.3 g COD/L (5^th^ day), 4.7 g COD/L (4^th^ day), and 3.0 g COD/L (2^nd^ day), respectively. Moreover, acetic acid and butyric acid were the dominant products of VFAs. The maximum concentrations of butyric acid (2.8 g COD/L) and propanoic acid (0.6 g COD/L) were obtained from the substrate pretreated by AC at pH 3 on the 5^th^ day of fermentation. *Romboutsia* species have this ability to produce butyric acid, and according to microbiology results, the higher percentage of this species was found in a sample pretreated by AC at pH 3, while the lowest percentage was found in a sample pretreated by AC at pH 11 [73].

#### 3.3.3 Evaluation of the combination of AC with H_2_O_2_ in the pretreatment stage

Advanced oxidation processes (AOPs) are useful techniques working through the formation of reactive radicals, capable of attacking target molecules. These radicals have the potential to oxidize complex molecules and produce simpler ones [6,74,75]. In this study, hydrogen peroxide (H_2_O_2_) at a concentration of 500 ppm was combined with AC at a frequency of 24 kHz, power density of 400 W/L, and substrate loading of 5 % w/v in 30 min as a pretreatment followed by fermentation. The outcomes of this hybrid process were compared to the individual ones to investigate the influence of the combined process on the magnitude of sCOD and the concentration of LA and VFAs. Rice combined with H_2_O_2_ was kept on a stirrer at a speed of 500 rpm for 30 min to determine the result of sole H_2_O_2_.

H_2_O_2_ is regarded as an oxidant that is capable of the degradation of various pollutants [22]. Cavitation combined with H_2_O_2_ leads to the formation of hydroxyl radicals (^•^OH) according to Eq. (9).

(9) H_2_O_2_ +AC → 2^•^OH

The effect of the combination of this agent with AC on the solubility represented by sCOD is shown in Table 4.

**Table 4:** Effect of combination of AC with H_2_O_2_ in the pretreatment stage on sCOD at a frequency of 24 kHz, power density of 400 W/L, substrate loading of 5 % w/v, H_2_O_2_ concentration of 500 ppm, and AC duration of 30 min.

**Pretreatment process**	***[H_2_O_2_] (ppm)***	**Stirrer speed (rpm)**	**VSS (g/L)**	**Increase in sCOD%**
**AC**	***−***	−	14.99 ± 0.12	555 ± 17
**AC + H_2_O_2_**	***500***	−	14.91 ± 0.21	615 ± 31
**H_2_O_2_**	***500***	500	15.26 ± 0.19	85 ± 11

In H_2_O_2_-based AOPs, hydroxyl radicals break down sludge by degrading its structure and destroying cell walls, which can result in the release of intracellular materials and the conversion of extracellular polymeric substances into soluble organic compounds [76]. In the case of the disintegration of rice content, almost a small difference in sCOD was observed between sole AC and AC combined with H_2_O_2_. It might be related to the magnitude of mechanical effects induced by AC, which can decrease the importance of free radicals provided by the low concentration of H_2_O_2_ for the disintegration of starch.

**Fig. 9:**
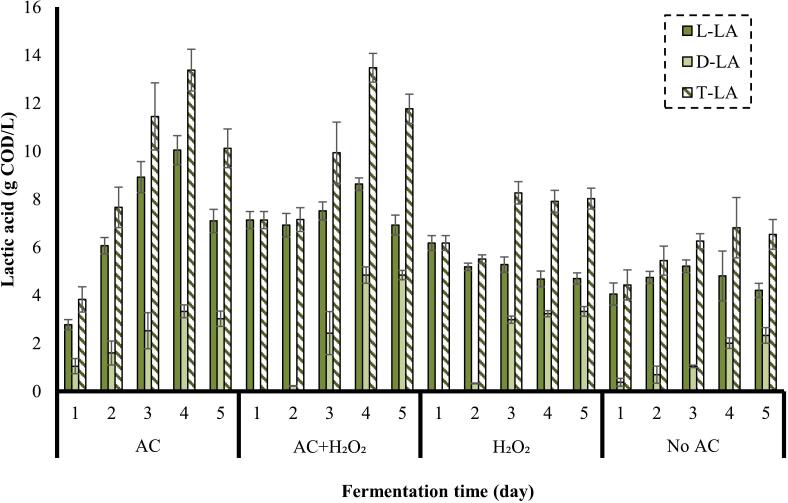
Effect of the combination of AC with H_2_O_2_ in the pretreatment stage on the concentration of LA (frequency: 24 kHz, power density: 400 W/L, substrate loading: 5 % w/v, [H_2_O_2_]: 500 ppm, AC duration: 30 min).

The effect of H_2_O_2_ on the production of LA was investigated and the results are demonstrated in Fig. 9. A low concentration of H_2_O_2_ (500 ppm) for 5 % w/v rice was selected to avoid the adverse effect of H_2_O_2_ on the microorganisms in the fermentation process. However, in the future, the different concentrations of H_2_O_2_ in different pH should be investigated to determine the effect of H_2_O_2_ more accurately.

The maximum concentration of L-LA (10 g COD/L) was obtained from the substrate pretreated by sole AC, which was higher than the maximum concentration achieved from the substrate pretreated by the combination of H_2_O_2_ and AC (8.6 g COD/L). The reason behind this decrease might be the lower percentage of *Limosilactobacillus* sp. in the substrate pretreated by AC + H_2_O_2_. Nevertheless, the combination of H_2_O_2_ and AC led to the highest concentration of D-LA (4.8 g COD/L). The highest value might be related to the highest percentage of *Lactobacillus* sp.*,* which have the ability to produce D-LA [70]. The substrate pretreated by sole H_2_O_2_ produced higher amount of L-LA (6.1 g COD/L) and D-LA (3.3 g COD/L) in comparison with the control process. It is noticed that AC led to the production of more L-LA compared to H_2_O_2_ + AC, however, H_2_O_2_ + AC resulted in the formation of more D-LA compared to sole AC. This is the reason that the maximum T-LA amounts obtained from the substrates pretreated by AC and AC + H_2_O_2_ were almost the same. According to the results, it is worth mentioning that the substrates pretreated in the presence of H_2_O_2_ led to the faster production of L-LA. The amounts of L-LA produced on the 1^st^ day of fermentation from the substrates pretreated by AC + H_2_O_2_ and sole H_2_O_2_ were higher compared to sole AC and the control process.

**Fig. 10:**
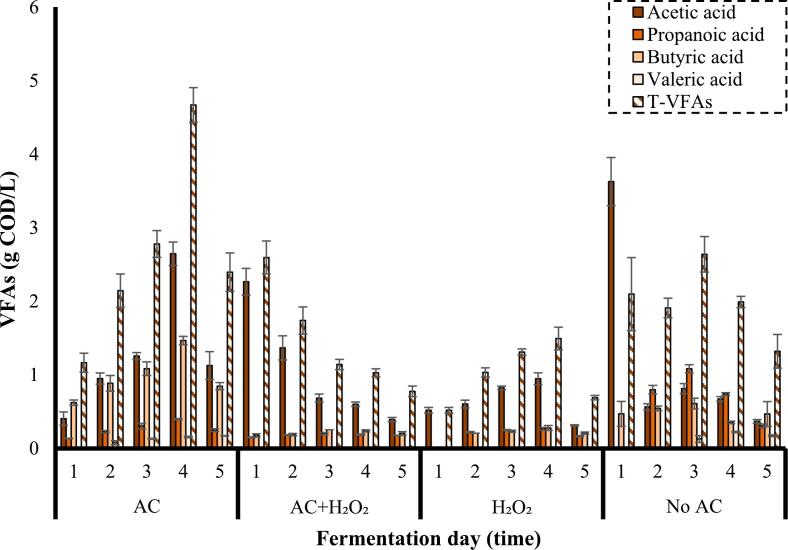
Effect of the combination of AC with H_2_O_2_ in the pretreatment stage on the concentration of VFAs (frequency: 24 kHz, power density: 400 W/L, substrate loading: 5 % w/v, [H_2_O_2_]: 500 ppm, AC duration: 30 min).

The effect of H_2_O_2_ on the production of VFAs is observed in Fig. 10.

The concentration of VFAs produced from the substrate pretreated by the combination of AC and H_2_O_2_ shows that the presence of this oxidant reduced the production of VFAs. VFAs concentration peaked at 2.6 g COD/L by the application of AC + H_2_O_2_ on the 1^st^ day and 1.5 g COD/L by sole H_2_O_2_ on the 4^th^ day. Moreover, acetic acid was the dominant product of VFAs in the presence of H_2_O_2_. It was hypothesized that the presence of H_2_O_2_ might have completely disabled the microorganism producing valeric acid and drastically hindered the microorganisms which were responsible for the production of butyric acid.

### 3.4 Economic assessment

The economic viability of a pretreatment process like AC is closely tied to its ability to intensify the yield of value-added products and reduce waste volumes as a way to improve waste management [77]. While pretreatment methods offer important benefits such as improving substrate bioavailability, reducing inhibitory compounds, and enhancing process efficiency, they introduce extra operational expenses. The application of acoustic cavitation systems as a pretreatment stage for conventional fermentation processes increases operating costs. Consequently, an economic evaluation is essential to determine the viability of implementing such a pretreatment approach.

The cost of electricity for industrial use in Poland is 83.74 EUR/ MWh reported on May 2025 [78]. Currently, the industrial prices for hydrogen peroxide (H_2_O_2_), sodium hydroxide (NaOH), and hydrochloric acid (HCl) are approximately 0.54, 0.25, and 0.11 US dollar/kg in Europe (they were converted to EUR/kg for the calculation), respectively [[79], [80], [81]]. The average price of LA is approximately 4 EUR/kg in Europe [82].

The highest amount of T-LA achieved by the conventional fermentation without any pretreatment (control process) was 6.4 g/L observed on the 4^th^ day of fermentation. While, the highest T-LA obtained from the substrates pretreated by the utilization of low-frequency AC at power densities of 400 W/L and 300 W/L for 30 min (total energy consumption of 0.2 kWh and 0.15 kWh, respectively) was 12.50 g/L (6.1 g/L increase compared to the control process) and 9.5 g/L (3.1 g/L increase), respectively. Additionally, the substrate pretreated by the combination of H_2_O_2_ (500 ppm) with a low-frequency AC at a power density of 400 W/L for 30 min has caused the maximum T-LA production of 12.6 g/L. The highest T-LA achieved from the substrate pretreated by the combined process of AC (24 kHz, 400 W/L) and HCl (pH 3) was 11.6 g/L. Furthermore, the highest T-LA obtained from the substrate pretreated by the combination of AC (24 kHz, 400 W/L) and NaOH (pH 11) was 12.2 g/L.

In Table 5, the summary of the economic assessment was reported for the production of LA as the main product of this study. This assessment investigated the net saving obtained as a result of maximum T-LA obtained from the substrate pretreated by sole AC at a power density of 400 W/L and 300 W/L, and AC combined with H_2_O_2_, NaOH, and HCl, compared to the conventional fermentation. The values observed in the table were calculated per one ton of substrate treatment.

**Table 5:** Economic assessment for AC pretreatment processes compared to the conventional fermentation per ton of substrate treatment (frequency: 24 kHz, [H_2_O_2_]: 500 ppm, AC duration: 30 min).

**Pretreatment process**	**Energy input (kWh)**	**Energy input (€)**	**Additive (€)**	**Increase in LA (kg)**	**Increase in LA (€)**	**Net saving compared** **to the control (€)**
AC (300 W/L)	3000	251	−	62	248	−3
AC (400 W/L)	4000	335	−	122	488	***153***
AC + H_2_O_2_	4000	335	4.8	124	496	***156***
AC + NaOH (pH 11)	4000	335	0.18	116	464	129
AC + HCl (pH 3)	4000	335	0.07	104	416	81

Net saving compared to control (€) = Increase in total LA (€) compared to the conventional fermentation– Energy input (€) – additives (€).

According to this assessment, the combination of AC + H_2_O_2_ at a H_2_O_2_ concentration of 500 ppm can be regarded as an economically viable technique, leading to a net saving of €156 per ton of substrate treatment. Sole AC under optimum conditions (24 kHz, 400 W/L) can result in a net savings of €153 per ton of substrate. In addition, it still has the potential to increase additional financial benefits related to other valuable products formed during the fermentation such as VFAs. Nevertheless, this factor is excluded from the current economic evaluation.

The pretreatment set-up also raises the total initial costs, and the reduction in the amount of solid processed by the pretreatment method can decrease the cost of dewatering, transportation, and landfill, however, this analysis does not take those factors into account.

## 4 Conclusions

AC pretreatment has proven its ability to elevate the concentration of soluble compounds, enhancing their availability to microorganisms involved in the fermentation. At the same power density (300 W/L), the low-frequency (24 kHz) reactor showed significantly better performance in increasing sCOD. The higher production of hydroxyl radicals in the low-frequency reactor was confirmed using p-CBA as a probe compound for hydroxyl radicals as well as employing potassium iodide. Increasing power density from 100 W/L to 400 W/L led to a continuous increase in sCOD and L-LA production. The application of low frequency AC at a power density of 400 W/L enhanced sCOD by 555 %, L-LA by 92 %, D-LA by 43 %, and T-VFAs by 15 %.

Rice pretreated with the combination of AC and acid (HCl) resulted in 750 % increase in sCOD, 100 % increase in D-LA, and 29 % increase in T-VFAs. Pretreatment by the combination of AC and alkali (NaOH) also increased sCOD and D-LA by 625 % and 104 %, respectively. Additionally, combining AC with H_2_O_2_ increased D-LA by 109 %. Acetic acid and butyric acid were the dominant VFAs under all investigated conditions.

Microbiological analysis demonstrated that *Limosilactobacillus* sp. dominated in all digestate samples, indicating its key role in LA production. The highest abundance of this species was observed in the sample pretreated by sole AC. The highest abundance of *Lactobacillus* sp.*,* producing D-LA, was found in the samples pretreated by AC + H_2_O_2_. In contrast, the lowest abundance of this species was observed in the sample pretreated by sole AC.

An economic assessment for the production of LA as the main product revealed that using sole AC (400 W/L, 24 kHz, 30 min) and the combination of AC and H_2_O_2_ (400 W/L, 24 kHz, [H_2_O_2_]: 500 ppm, 30 min) could lower operating expenses by €153 and €156 per the ton of substrate treatment, respectively, compared to conventional fermentation processes without pretreatment.

Future studies could further improve this method by optimizing the use of additives to further enhance microbial access to nutrients and adjust microbial community dynamics. Moreover, exploring cavitation-based processes across various types of food waste may reveal their broader applicability, promoting bioresource utilization in diverse settings. Future research should include process upscaling and the application of cavitation continuous-flow systems. This detailed study sets a pathway for the selection and application of modern pretreatment techniques in bioengineering, potentially influencing future studies on renewable energy production and bioproduct synthesis.

## CRediT authorship contribution statement

**Zahra Askarniya:** Writing – original draft, Visualization, Methodology, Investigation, Data curation, Conceptualization. **Aisha Khan Khanzada:** Writing – original draft, Visualization, Investigation, Data curation. **Sławomir Ciesielski:** Writing – original draft, Investigation. **Zongsu Wei:** Writing – review & editing, Validation. **Jacek Mąkinia:** Writing – review & editing, Supervision, Project administration, Funding acquisition. **Grzegorz Boczkaj:** Writing – review & editing, Writing – original draft, Validation, Supervision, Project administration, Methodology, Conceptualization.

## Declaration of competing interest

The authors declare that they have no known competing financial interests or personal relationships that could have appeared to influence the work reported in this paper.
